# Astroglial Cx30 sustains neuronal population bursts independently of gap‐junction mediated biochemical coupling

**DOI:** 10.1002/glia.23591

**Published:** 2019-02-22

**Authors:** Ulrike Pannasch, Elena Dossi, Pascal Ezan, Nathalie Rouach

**Affiliations:** ^1^ Neuroglial Interactions in Cerebral Physiopathology Center for Interdisciplinary Research in Biology, Collège de France, Centre National de la Recherche Scientifique UMR 7241, Institut National de la Santé et de la Recherche Médicale U1050, Labex Memolife, PSL Research University Paris France

**Keywords:** astrocyte, connexin 30, gap junctions, glutamate transporter, hippocampus, neuronal population bursts

## Abstract

Astroglial networks mediated by gap junction channels contribute to neurotransmission and promote neuronal coordination. Connexin 30, one of the two main astroglial gap junction forming protein, alters at the behavioral level the reactivity of mice to novel environment and at the synaptic level excitatory transmission. However, the role and function of Cx30 at the neuronal network level remain unclear. We thus investigated whether Cx30 regulates neuronal population bursts and associated convulsive behavior. We found in vivo that Cx30 is upregulated by kainate‐induced seizures and that it regulates in turn the severity of associated behavioral seizures. Using electrophysiology ex vivo, we report that Cx30 regulates aberrant network activity via control of astroglial glutamate clearance independently of gap‐junction mediated biochemical coupling. Altogether, our results indicate that astroglial Cx30 is an important player in orchestrating neuronal network activity.

## 1 INTRODUCTION

Rhythmic, synchronized bursting of neuronal ensembles is a hallmark of neuronal activity in the healthy and diseased brain. Astrocytes are attractive candidates for orchestrating neuronal populations. They are indeed part of the tripartite synapse, where they integrate and modulate ongoing neuronal transmission (Perea, Navarrete, & Araque, 2009) via various mechanisms, including metabolic supply, gliotransmitter release, or contact‐mediated signaling (Araque et al., 2014; Bernardinelli, Muller, & Nikonenko, 2014; Clarke & Barres, 2013; Dallérac, Chever, & Rouach, 2013; Rouach, Koulakoff, Abudara, Willecke, & Giaume, 2008). Astrocytes are also organized in large, communicating networks that are compartmentalized and superposed to local neuronal ensembles. Astroglial networks are mediated by gap junction channels, formed by connexins (Cxs), which provide the structural basis for an extensive intercellular communication. Gap‐junction astroglial networks contribute to neurotransmission by both, fueling metabolic active synapses with proper nutrients (Rouach et al., 2008) and by preventing excessive synaptic activity through control of extracellular homeostasis (Pannasch et al., 2011; Pannasch, Derangeon, Chever, & Rouach, 2012; Wallraff, 2006). In addition, astroglial networks also control neuronal population bursts (Pannasch et al., 2012; Rouach et al., 2008) and promote neuronal coordination (Chever, Dossi, Pannasch, Derangeon, & Rouach, 2016). Remarkably, gap junction channels in astrocytes are formed by two Cx isoforms, Cx43 and Cx30, and recent data have unraveled an unexpected complexity of each Cx, which present both channel (gap junction and hemichannel) and non‐channel functions, including protein interactions, cell adhesion and intracellular signaling (Pannasch & Rouach, 2013).

While astroglial Cx43 is a well‐known regulator of neuronal physiology through channel and non‐channel functions (Chever, Lee, & Rouach, 2014; Chever, Pannasch, Ezan, & Rouach, 2014; Clasadonte, Scemes, Wang, Boison, & Haydon, 2017; Meunier et al., 2017; Retamal & Sáez, 2014; Roux et al., 2015; Samoilova, Wentlandt, Adamchik, Velumian, & Carlen, 2008; Stehberg et al., 2012; Theis et al., 2003; Wang, Xu, Wang, Takano, & Nedergaard, 2012), the contribution of Cx30 to physiological and pathological neuronal activity as well as associated behavior remains poorly investigated. At the behavioral level, Cx30 alters the reactivity of mice to novel environments and object recognition memory (Dere et al., 2003). At the synaptic level, Cx30 tunes hippocampal excitatory synaptic transmission by determining the efficacy of astroglial glutamate clearance through an unprecedented regulation of astroglial morphology independent of gap‐junction‐mediated biochemical coupling. In fact, by regulating astroglia ramification and the extent of astroglial processes contacting synaptic clefts, Cx30 directly sets synaptic glutamate levels through clearance (Pannasch et al., 2014). Cx30 is thus a molecular determinant of astroglial synapse coverage controlling synaptic efficacy. In all, astroglial Cx30 functions are complex and can either promote or dampen synaptic transmission. However, what is the specific role of Cx30 on neuronal network activity and which function of Cx30 is involved are unknown. We, thus, investigated whether Cx30 favors or limits aberrant neuronal network activity and associated convulsive behavior. We here found in vivo that Cx30 expression is increased by kainate‐induced seizures and regulates in turn the associated convulsive behavior in mice. Accordingly, we show ex vivo that Cx30 modulates aberrant neuronal population bursts induced by increased excitability via control of astroglial glutamate uptake independently of gap‐junction mediated biochemical coupling.

## 2 MATERIALS AND METHODS

Experiments were carried out according to the guidelines of the European Community Council Directives of January 1st 2013 (2010/63/EU) and of the local animal welfare committee (certificate A751901, Ministère de l'Agriculture et de la Pêche), and all efforts were made to minimize the number of animals used and their suffering. Cx30^−/−^ (−/−) mice were generated as previously described (Teubner et al., 2003) and provided by K. Willecke, University of Bonn, Germany. C57Bl6 (+/+) mice were supplied by Charles River, France. Heterozygous mice carrying the knockout mutation were interbred to obtain homozygous strain. Cx30^T5M/T5M^ mice (T5M; [Schütz et al., 2010]) were provided by F. Mammano, Venetian Institute of Molecular Medicine, Italy. For all analyses, mice of both genders and littermates were used.

### 2.1 In vivo kainate injections

The +/+ and −/− mice were injected i.p. with kainic acid (25 mg/kg) as previously described (Hu, Koh, Torgerson, & Cole, 1998). Mice were video‐monitored for 2 hr after seizure onset. Seizure severity was defined by grade 0 to IV (Ono, Vieth, & Walson, 1990). Grade 0: no response. Grade I: staring, nodding, rearing. Grade II: staring, nodding, rearing, front‐ or hind limb pawing. Grade III: staring, nodding, rearing, bilateral pawing, wobbling, falling. Grade IV: status epilepticus and death.

### 2.2 Immunoblot of Cx30 expression

Immunoblotting and quantification were performed as previously described (Pannasch et al., 2014). Hippocampi were collected in a small volume of cold SDS 2% containing a cocktail of protease inhibitors and phosphatase inhibitors (β‐glycerophosphate (10 mM) and orthovanadate (1 mM)) to which Laemmli 5× buffer was added. Samples were sonicated, boiled 5 min and loaded on 4–12% polyacrylamide gels. Equal amounts of proteins were separated by electrophoresis and transferred onto nitrocellulose membranes. Membranes were saturated with 5% fat‐free dried milk in triphosphate buffer solution and incubated overnight at 4 °C with primary antibodies (GAPDH rabbit monoclonal antibody [Sigma], Cx30 rabbit polyclonal antibody). They were then washed and exposed to peroxidase‐conjugated secondary antibodies (donkey anti‐rabbit IgG HRP‐conjugated secondary antibodies, Amersham Biosciences). GAPDH was used as loading control. Specific signals were revealed with the chemiluminescence detection kit (ECL, GE Healthcare). Semi‐quantitative densitometric analysis was performed after scanning the bands with the imageJ software.

### 2.3 Ex vivo electrophysiology

Acute transverse hippocampal slices (400 μm) were prepared as previously described (Chever et al., 2016) from 20 to 25 days‐old wild type (+/+), Cx30 knockout (−/−) (Teubner et al., 2003), and Cx30 T5M mice (Schütz et al., 2010). Slices were maintained at room temperature in a storage chamber that was perfused with an artificial cerebrospinal fluid (ACSF) containing (in mM): 119 NaCl, 2.5 KCl, 2.5 CaCl_2_, 1.3 MgSO_4_, 1 NaH_2_PO_4_, 26.2 NaHCO_3_, and 11 glucose, saturated with 95% O_2_ and 5% CO_2_, for at least 1 hr prior to recording. Slices were transferred to a submerged recording chamber mounted on an Olympus BX51WI microscope equipped for infrared‐differential interference (IR‐DIC) microscopy and were perfused with ACSF at a rate of 1.5 ml/min at room temperature. Extracellular field and whole‐cell patch‐clamp recordings were performed. Stratum radiatum astrocytes were identified by their small cell bodies, low input resistance (~20 MΩ), high resting potentials (~ −80 mV) and linear IV curves. Field excitatory postsynaptic potentials (fEPSPs) were recorded from 400 μm slices with glass pipettes (2–5 MΩ) filled with ACSF and placed in *stratum radiatum*. Stimulus artifacts were blanked in sample traces. Hippocampal population bursts were either induced by removal of Mg^2+^ and inhibition of GABAergic transmission by 100 μM Picrotoxin (0 Mg‐Picro) at least 1 hr prior to recording or by acute perfusion of slices with ACSF containing 200 μM BaCl_2_. Somatic whole‐cell recordings were obtained from visually identified CA1 *stratum radiatum* astrocytes, using 5–10 MΩ glass pipettes filled with (in mM): 105 K‐gluconate, 30 KCl, 10 HEPES, 10 phosphocreatine, 4 ATP‐Mg, 0.3 GTP‐Tris, and 0.3 EGTA (pH 7.4, 280 mOsm). Glutamate transporter currents were evoked synaptically by stimulation of Schaffer collaterals (0.1 Hz) with ACSF filled glass pipettes and were recorded simultaneously with fEPSPs. The field recording pipette was placed 50 μm away from the recorded astrocyte and transporter currents were blocked either by DL‐threo‐β‐Benzyloxyaspartatic acid (TBOA, 200 μM). GLT‐1 activity was partially inhibited by acute perfusion with 50 μM DHK. Recordings were acquired with Axopatch‐1D amplifiers (Molecular Devices, San Jose, CA), digitized at 10 kHz, filtered at 2 kHz, stored and analyzed on computer using Pclamp9 and Clampfit9 software (Molecular Devices).

### 2.4 Immunohistochemistry

Saline and KA‐injected mice were perfused with phosphate buffered saline (PBS) 4 hr after injection and their brain rapidly removed and frozen. Cryostat brain slices were then cut and fixed for 10 min at room temperature with 4% paraformaldehyde (PFA), washed three times with PBS and pre‐incubated 1 hr with PBS‐1% gelatin in the presence of 1% Triton‐X100. Brain slices were then immunostained overnight at 4 °C for GFAP (1:500, mouse anti‐GFAP antibody, Sigma‐Aldrich) and Cx30 (1:500, rabbit anti‐Cx30 antibody, ThermoFisher) and washed in PBS three times. Appropriate secondary antibodies (goat anti‐mouse IgG conjugated to Alexa 488 and goat anti‐rabbit IgG conjugated to Alexa 561, 1:200, ThermoFisher) were finally applied for 1–2 hr at room temperature, followed by DAPI staining (1:2,000, ThermoFisher). After several washes, brain slices were mounted in fluoromount (Southern Biotechnology) and examined with a spinning‐disk confocal microscope (Eclipse *Ti*, Nikon) equipped with CMOS camera (Photometrics). Stacks of consecutive images were taken with a 60× objective at 500 nm intervals and acquired sequentially with 3 lasers (405, 488, and 561 nm). Z‐projections were then reconstructed using ImageJ software and average fluorescence intensity for Cx30 was measured in hippocampal CA1 *stratum radiatum* astrocytes.

### 2.5 Statistics

All data are expressed as mean ± *SEM*. Statistical significance for between‐group comparisons was determined by unpaired or paired *t*‐tests. Fisher exact test was used to compare distributions.

### 2.6 Drugs

Picrotoxin was obtained from Sigma and all other drugs were from Tocris.

## 3 RESULTS

### 3.1 Cx30 expression is increased by kainate and regulates convulsive behavior

**Figure 1:**
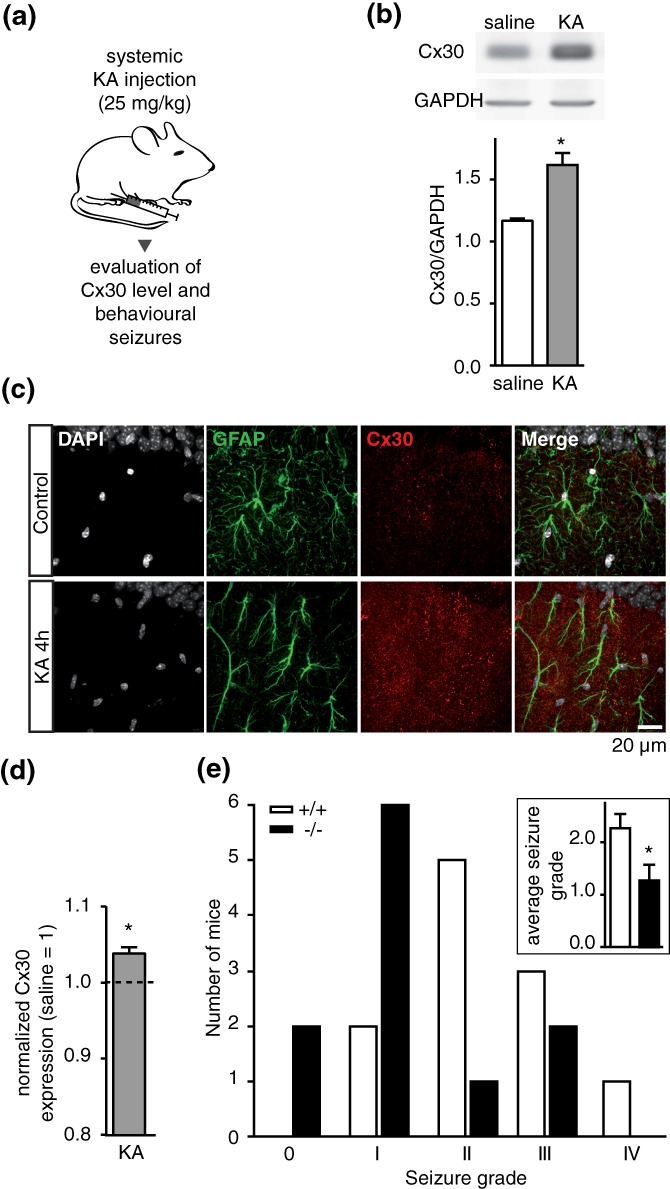
Cx30 levels are increased by kainic acid and regulate behavioral seizures. (a) Schematic representation of the experimental protocol: Mice are systemically injected with kainate (25 mg/kg, i.p). Within 2–4 hr after injection, Cx30 levels and behavioral seizures are evaluated. (b) Top, representative Cx30 immunoblot analysis of hippocampal extracts from saline and kainate injected wildtype mice. Bottom, quantification of Cx30 levels in saline (*n* = 4) and kainate (*n* = 4) injected mice shows increased Cx30 expression after KA injection (*p* < 0.05). (c) Representative confocal images of CA1 *stratum radiatum* showing immunolabeling of astrocytes (GFAP, green), Cx30 (red), and nuclei (DAPI, gray) in control and KA‐injected mice (4 hr after injection). Scale bar: 20 μm. (d) Quantification of Cx30 levels in astrocytes by confocal microscopy. Data are normalized to control (saline injection). (e) Analysis of kainate‐induced seizure grade in +/+ (*n* = 11) and −/− (*n* = 11) mice. Seizure grades were defined during the first 2 hr after i.p. injection of 25 mg/kg kainic acid. Grade 0: No response. Grade I: Staring, nodding, and rearing. Grade II: Staring, nodding, rearing, front‐ or hind limb pawing. Grade III: Staring, nodding, rearing, bilateral pawing, wobbling, and falling. Grade IV: Status epilepticus and death. Inset, quantification of total average seizure grade in +/+ (*n* = 11) and −/− (*n* = 11) mice. Asterisks indicate statistical significance (*p < 0.05) [Color figure can be viewed at wileyonlinelibrary.com]

Astrocytes regulate neuronal synchronization (Chever et al., 2016; Fellin et al., 2009; Lee et al., 2014; Poskanzer & Yuste, 2011; Sasaki et al., 2014; Wang et al., 2012), but the underlying molecular mechanisms remain elusive. Cx30 is an important player in the regulation of synaptic activity through various mechanisms (Chever et al., 2016; Pannasch et al., 2011, 2012, 2014). However, whether Cx30 is endogenously regulated by sustained network activity and regulates in turn such activity is currently unknown. To investigate whether Cx30 expression is dynamically regulated by synchronized network activity, we induced acute seizures in mice by injecting kainate (KA, 25 mg/kg i.p.; Figure 1a), a proconvulsant agent. We found an upregulation of Cx30 expression by approximately 40% (n = 4, *p* < 0.05) in the hippocampus of all KA‐injected wild type mice when compared with saline injection within 4 hr after injection (Figure 1b), while no significant change occurred at shorter time points (Supporting Information Figure S1). We also performed immunostaining for Cx30 in hippocampal CA1 area of control and KA‐injected mice 4 hr after injection (Figure 1c) and found a significant increase of Cx30 expression in *stratum radiatum* astrocytes (Figure 1d). We then assessed whether Cx30 regulates in vivo kainate‐induced behavioral seizures susceptibility and severity. Wildtype mice (+/+) and mice deficient for Cx30 (−/−) did not show spontaneous seizures before kainate injection. Behavioral seizures were induced within 15–20 min after kainic acid systemic injection (25 mg/kg) in all +/+ mice (n = 11), as previously described (Hu et al., 1998), and in the vast majority of −/− mice (82%; n = 11; Figure 1c), indicating similar susceptibility to seizures. However, the severity of the convulsive behavior was decreased in −/− mice (Figure 1c), which displayed mostly short grade 1 seizures (~55%; total average seizure grade: 1.27 ± 0.30, n = 11), in contrast to +/+ mice, which presented predominantly (~73%) grade 2–3 seizures (total average seizure grade: 2.27 ± 0.27, n = 11; *p* < 0.05). These data indicate that Cx30 deficiency decreases behavioral seizures severity.

### 3.2 Cx30 controls neuronal population bursts

**Figure 2:**
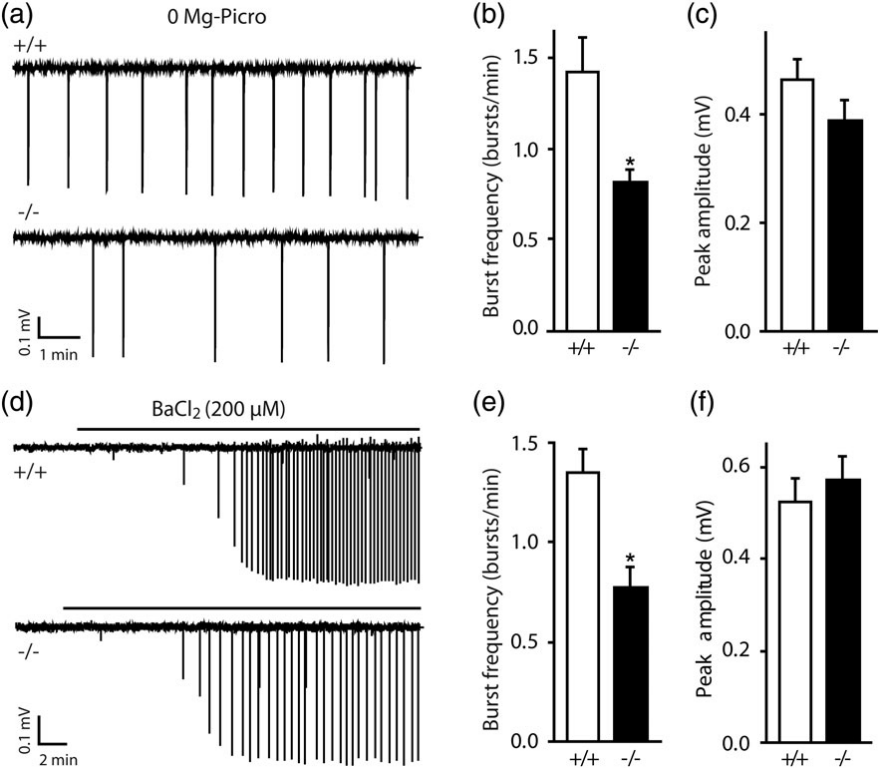
Hippocampal neurons show reduced neuronal population bursts in Cx30^−/−^ mice. (a–c) Burst frequency in hippocampal slices bathed in 0 mg‐Picro is reduced (*p* < 0.005; a,b) in −/− mice compared with +/+ mice (burst frequency: *n* = 28 and *n* = 24, respectively). Scale bars: 0.1 mV, 1 min. The burst peak amplitude was comparable in both genotypes (−/−: *n* = 36, +/+: *n* = 24; c). (d–f) Inhibition of potassium channels by BaCl_2_ (200 μM) reduced the number of bursts in −/− mice (*p* < 0.05, n = 5) in comparison to +/+ mice (*n* = 6; d,e). Scale bars: 0.1 mV, 2 min. Burst amplitude was similar in −/− (*n* = 5) and +/+ mice (*n* = 7; f). Asterisks indicate statistical significance (*p < 0.05)

To investigate whether Cx30 alters neuronal network activity, we recorded neuronal population bursts in the hippocampal CA1 area. Aberrant bursting activity was induced in disinhibited hippocampal slices by inhibition of GABA_A_ receptors with picrotoxin and removal of extracellular Mg^2+^ (0 Mg‐Picro), and was observed in the majority of wildtype slices (86%, n = 28). In contrast, only 64% of the slices (n = 56; *p* < 0.05) from −/− mice displayed neuronal population bursts. Furthermore, once induced, population bursts were less frequent (~ −43%) in −/− slices (*p* < 0.005, 0.8 ± 0.1 bursts/min, n = 28) compared with +/+ slices (1.4 ± 0.2 bursts/min, n = 24; Figure 2a,b), while their amplitude was unchanged (−/−: 0.39 ± 0.04 mV, n = 36; +/+: 0.46 ± 0.04 mV, n = 24; Figure 2a,c).

To evaluate whether such effect also occurs in a context of intact GABAergic transmission, we evoked bursting activity by inhibiting K^+^ channels with BaCl_2_ (200 μM, [Kivi et al., 2000]; Figure 2d). Whereas population bursts occurred in 64% of the slices (n = 11) from wildtype mice, only a minority of −/− slices (28%, n = 18) displayed such activity. As found in the previous model of aberrant network activity (0 Mg‐Picro), burst frequency was reduced in −/− slices (*p* < 0.05, 0.8 ± 0.1 burst/min, n = 5) compared with +/+ slices (1.8 ± 0.3 burst/min, n = 6, Figure 2d,e), whereas their amplitude was similar (−/−: 0.57 ± 0.04 mV, n = 5; +/+: 0.51 ± 0.04 mV, n = 6; Figure 2f).

**Figure 3:**
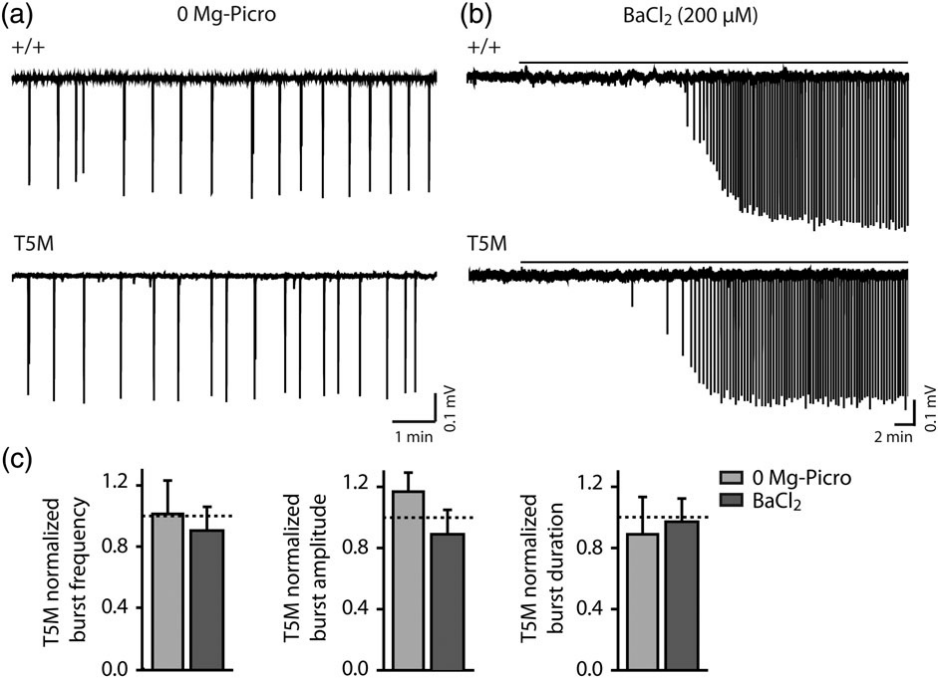
Cx30‐mediated impairment of bursting activity is not dependent on gap junction‐mediated biochemical coupling. Representative traces of burst firing induced by 0 mg‐Picro ACSF (a) or by BaCl_2_ (200 μM) treatment in normal ACSF (b) in hippocampal slices from +/+ and T5M mice. Scale bars: in a, 0.1 mV, 1 min; in b, 0.1 mV, 2 min. (c) Quantification of T5M burst frequency, amplitude and duration (normalized to +/+ values) after 0 mg‐Picro incubation (light gray) and BaCl_2_ treatment (dark gray). The two protocols induced bursts with similar frequency, amplitude and duration in +/+ and T5M mice (0 mg‐Picro: +/+, *n* = 9; T5M, *n* = 9; BaCl_2_: +/+, *n* = 10; T5M, *n* = 11)

Astroglial Cx30 modulates basal hippocampal synaptic transmission independently of intercellular biochemical coupling. We thus investigated neuronal population discharges in hippocampal slices from Cx30 T5M mice, in which the replacement of a threonine by a methionine at position 5 of Cx30 results in a defective Cx30 channel pore but intact membrane targeting (Grifa et al., 1999; Schütz et al., 2010). The pattern of neuronal bursts induced in the two models of aberrant network activity (0 Mg‐Picro and BaCl_2_), as described above, were comparable in T5M and +/+ mice (Figure 3a,c).

Altogether, these data suggest that Cx30 promotes neuronal bursts independently of gap‐junction mediated biochemical coupling.

### 3.3 Enhanced glial glutamate clearance dampens neuronal network activity in Cx30^−/−^ mice

**Figure 4:**
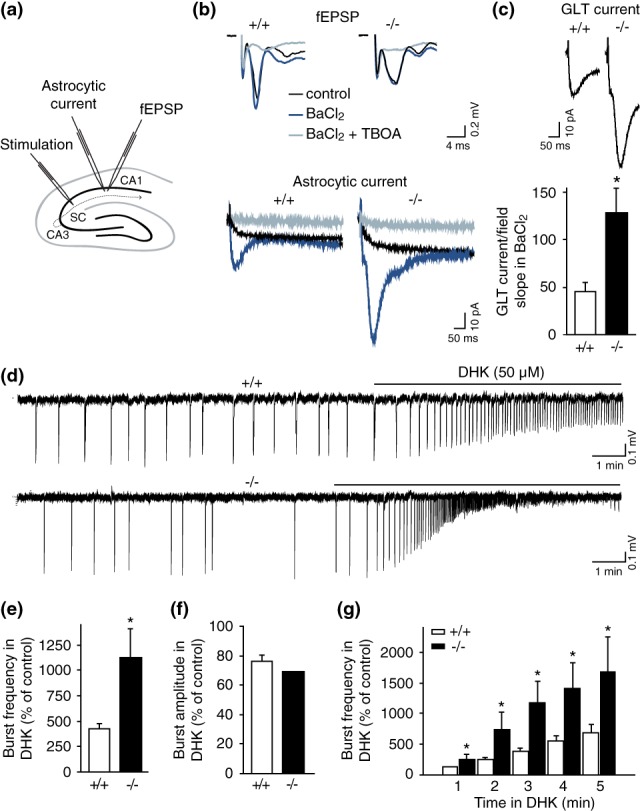
Enhanced glial glutamate uptake reduces aberrant bursting activity in Cx30 deficient mice. (a) Scheme of the hippocampal slice illustrating in the CA1 area the position of the stimulating electrode to activate the Schaffer collaterals (SC), the patch pipette electrode to record astrocytic currents, and the extracellular electrode. (b) Pharmacological isolation of astroglial glutamate transporter currents evoked by Schaffer collateral stimulation in +/+ (left) and −/− (right) mice. Simultaneous recordings of neuronal field potential (top) and astroglial whole‐cell currents (bottom) in response to a single SC stimulation are shown in control (black traces). Application of BaCl_2_ (200 μM), which inhibits K^+^ channels, increases neuronal response and unmasks the GLT component of astrocytic current (dark blue traces). In the presence of BaCl_2_ and TBOA (200 μM), an inhibitor of GLT, a slow current component remains (light gray traces). Subtraction of this remaining current (light gray) to BaCl_2_‐insensitive current (dark gray) isolates the GLT current. Scale bars: Top, 0.2 mV, 4 ms; bottom, 10 pA, 50 ms. (c) GLT currents are enhanced in −/− astrocytes (*p* < 0.05, *n* = 11) during BaCl_2_ induced high neuronal activity compared with +/+ astrocytes (*n* = 6). Representative current traces are shown above the graph. Scale bars: 10 pA, 50 ms. (d,e) Partial inhibition of glial glutamate uptake by DHK (50 μM) results in a more profound increase in burst firing in −/− mice (*p* < 0.05, *n* = 9) than +/+ mice (*n* = 13). Scale bars: 0.1 mV, 1 min. (f) DHK reduced the burst amplitude in both genotypes (−/−, *p* < 0.05; +/+, *p* < 0.01). (g) Differential effect of DHK in −/− slices persists over time. Asterisks indicate statistical significance (*p < 0.05) [Color figure can be viewed at wileyonlinelibrary.com]

Cx30 deficiency reduces basal synaptic activity independently of gap junction intercellular biochemical coupling via an increase in astroglial glutamate transport (Pannasch et al., 2014). We thus here investigated whether an increase in glutamate clearance from −/− astrocytes also occurs in a high regime of activity characterized by coordinated population bursts and whether such enhanced glutamate uptake inhibits aberrant bursting activity. To do so, we performed simultaneous recordings of synaptically‐evoked glutamate transporter (GLT) currents in astrocytes and neuronal responses (field excitatory synaptic potentials, fEPSPs) in the presence of BaCl_2_ (Figure 4a,b). Whereas BaCl_2_ increased neuronal activity by approximately 50% in both genotypes (+/+: *p* < 0.05, before BaCl_2_: 0.40 ± 0.04, during BaCl_2_: 0.62 ± 0.1, n = 6; −/−: *p* < 0.01, before BaCl_2_: 0.28 ± 0.03, during BaCl_2_: 0.39 ± 0.05, n = 11; Figure 4b), glutamate clearance was enhanced by approximately 180% in −/− astrocytes, as assessed by GLT current amplitudes normalized to neuronal activity (*p* < 0.05; GLT current/fEPSPslope: −/−: 127.7 ± 26.4, n = 11; +/+: 45.5 ± 9.9, n = 6; Figure 4b,c).

If the increased astrocytic glutamate clearance is indeed decreasing aberrant network activity in −/− mice, reduction of astroglial GLT currents should have a stronger effect on neuronal populations bursts from −/− mice than from +/+ mice. Partial inhibition of GLT1, the most abundant glial GLT in the hippocampus, by dihydrokainic acid (DHK, 50 μM) resulted in a more profound increase (~ +94%) in the frequency of bursts in −/− mice (*p* < 0.05, n = 9) compared with +/+ mice (n = 13, Figure 4d,e), while burst amplitude was similarly reduced by approximately 20–30% in both −/− and +/+ mice (69.4 ± 4.8% and 76.2 ± 4.0% of control, for −/− and +/+ mice respectively, Figure 4f). Such effect was consistently observed at different time points after DHK application (Figure 4g). Thus, enhanced astroglial glutamate clearance contributes to the reduction of bursting activity in −/− mice.

## 4 DISCUSSION

We here show in vivo that Cx30 expression is increased after kainic acid injection and that it regulates the severity of kainate‐induced behavioral seizures. Accordingly, we also found that Cx30‐deficient mice display ex vivo less frequent neuronal population bursts due to an enhanced glial glutamate clearance, which tunes down neuronal network activity. Interestingly, we observed that the effect on neuronal burst firing is independent from gap junction‐mediated biochemical coupling.

### 4.1 Dynamic regulation of Cx30 expression

We found that systemic kainic acid injection in mice induces a strong increase in Cx30 expression, in agreement with Condorelli et al. (2002), who showed and early and transient (within 6 hr) upregulation of Cx30 expression in several brain regions after intracerebroventricular injection of KA in rats. Furthermore, we also observed that Cx30 in turn controls kainic acid‐induced behavioral seizures by increasing their severity. The systemic injection of kainate in mice represents a model of status epilepticus. Our results thus suggest that Cx30 expression may be activity‐dependent. Interestingly, several physiological and pathological situations are associated with changes in Cx30 expression. A 2–14 days exposure to an enriched environment increases Cx30 gene expression in mice (Rampon et al., 2000); similarly, modafinil‐induced psychostimulation augments Cx30 at mRNA and protein levels and enhances gap junctional communication in cortical astrocytes without affecting Cx43 levels (Liu et al., 2013). Conversely, decreased Cx30 levels have been found during excitotoxic brain injury in reactive astrocytes located in the area of neuronal death (Koulakoff, Ezan, & Giaume, 2008) and during astrocyte transformation into highly motile glioma cells (Princen et al., 2001). Furthermore, patients with major depression disorders or suicide completers show decreased brain levels of Cx30 (Bernard et al., 2011; Ernst et al., 2011). Interestingly, a decrease in Cx30 expression has also been found 6–24 hr or 7 days after KA injection in rats (Condorelli et al., 2002; Takahashi, Vargas, & Wilcox, 2010). We can speculate that Cx30 expression is dynamically regulated during time and that an early (4–6 hr) initial increase of expression linked to seizure development after KA injection is followed by a decrease, which may correlate to tissue damage consequent to seizures. Altogether, these findings suggest that Cx30 expression and function could be regulated in an activity‐dependent manner. Consistent with this hypothesis, gap junction coupling mediated by astroglial Cx43, the other gap junction forming protein, has been reported to be dynamic as it is regulated by neuronal activity (Rouach, Glowinski, & Giaume, 2000; Rouach, Tence, Glowinski, & Giaume, 2002; Rouach et al., 2008; Roux, Benchenane, Rothstein, Bonvento, & Giaume, 2011).

### 4.2 Cx30‐mediated regulation of bursts and behavioral seizures

We have shown that Cx30, which is upregulated after kainate injection, worsen kainate‐induced behavioral seizures and regulates neuronal population bursts. These findings, together with physiological and pathological modulations of Cx30 expression (Condorelli et al., 2002; Koulakoff et al., 2008; Liu et al., 2013; Rampon et al., 2000; Roux et al., 2011), suggest reciprocal regulations between neuronal activity and astroglial Cx30. Indeed, enhanced neuronal activity may boost Cx30 expression, which would in turn intensify neuronal activity. This positive feedback loop could thus favor aberrant bursting ex vivo and increase the severity of seizures in vivo.

Interestingly, we have found that Cx30‐deficient mice display enhanced glial glutamate clearance, which contributes to decrease the frequency of hippocampal neuronal population bursts. Therefore, our results identify the Cx30‐mediated control of astroglial glutamate clearance as a critical factor controlling neuronal bursting activity. The enhanced astroglial glutamate transport may have the potential to tune the threshold for neuronal burst initiation and the recovery of neurons to the resting state by removing rapidly glutamate from synaptic and extrasynaptic sites. These effects most likely result from a reduction in glutamate receptor activation and the accompanied depolarization of neurons.

We previously showed that the increased glutamate clearance in Cx30 deficient mice results from changes in astrocytic morphology and coverage of synapses independently of gap junction‐mediated biochemical coupling. This leads structurally to a closer proximity of GLT1 to synaptic active zones and functionally to a depression of basal excitatory synaptic transmission (Pannasch et al., 2014). In the present study, revealing the role of Cx30‐mediated enhanced glutamate clearance in bursting activity independently of biochemical coupling, a similar mechanism is likely to be at play. Yet, we cannot exclude that Cx30 hemichannels also contribute to the control of bursting activity. However, although Cx30 can form hemichannels (Nielsen, Alstrom, Nicholson, Nielsen, & MacAulay, 2017), to date, their presence and functional activation have not been reported in astrocytes. Furthermore, there is presently no pharmacological tool to selectively and acutely inhibit their activity. These limitations hinder the study of their activation and selective implication in the regulation of neuronal activity. The development of specific Cx30 hemichannel blockers should allow future investigation of their contribution to bursting activity.

Finally, our in vivo results indicate that astroglial glutamate clearance play a crucial role in reducing the severity of seizures. Consistent with our data, a reduction in astroglial glutamate transporter expression associated with increased extracellular glutamate levels has been found in the sclerotic hippocampus of patients with temporal lobe epilepsy (Cavus et al., 2005, 2008; Jabs, Seifert, & Steinhäuser, 2008; Mathern et al., 1999; Proper et al., 2002) as well as in a tuberous sclerosis epilepsy model (Wong et al., 2003). Furthermore, knockout mice for the glial glutamate transporter GLT‐1 develop spontaneous seizures and hippocampal pathology resembling those observed in temporal lobe epilepsy patients with hippocampal sclerosis (Petr et al., 2015; Sugimoto et al., 2018; Tanaka et al., 1997). In addition, pharmacological block of GLT‐1 decrease the threshold to trigger epileptiform activity and increase the occurrence of spontaneous epileptiform discharges in the rat cortex (Campbell & Hablitz, 2004, 2005). Conversely, increase in GLT‐1 expression with the beta‐lactam antibiotic ceftriaxone (Rothstein et al., 2005) has already been shown to have anti‐convulsant effects (Jelenkovic et al., 2008). In all, these findings suggest that efficient astrocytic glutamate uptake by GLT‐1 may be essential to counteract epileptogenesis. Thus, disrupting astroglial Cx30 to enhance GLT‐1 activity could represent a novel therapeutic strategy against seizures.

## CONFLICT OF INTEREST

The authors declare that they have no conflict of interest.

## Supporting information


**Figure S1** Time‐dependent change of Cx30 expression following KA injection. (a) Representative Cx30 immunoblot analysis of hippocampal extracts from saline and kainate injected wildtype mice at 1 and 3 hr after injection. (b) Quantification of Cx30 levels at 1, 3, and 4 hr following i.p. KA injection (*n* = 4 for 1 and 4 hr and *n* = 3 for 3 hr). Data are normalized on control (saline injection, i.p.) (*p < .05).
